# Subcutaneous incision of the fistula tract and internal sphincterotomy (SIFT‐IS): a novel surgical procedure for transsphincteric anal fistula

**DOI:** 10.1111/codi.16297

**Published:** 2022-08-23

**Authors:** Rikisaburo Sahara, Michihiro Koizumi, Koji Morimoto, Itaru Kubota

**Affiliations:** ^1^ Proctology Center Makita General Hospital Tokyo Japan; ^2^ Nishiarai Coloproctology Clinic Tokyo Japan; ^3^ Department of Gastrointestinal and Hepato‐Biliary‐Pancreatic Surgery Nippon Medical School Tokyo Japan

**Keywords:** anal fistula, deep crypt, subcutaneous incision of the fistula tract and internal sphincterotomy, transsphincteric fistula

## Abstract

**Aim:**

The aetiology of anal fistula has not been fully clarified. One of the causes of anal fistulas may be the markedly deep crypts that characterize the primary openings. We developed subcutaneous incision of the fistula tract and internal sphincterotomy (SIFT‐IS) to eradicate these deep crypts. The aim of this study was to evaluate outcomes in patients with anal fistula treated with SIFT‐IS.

**Method:**

A retrospective study was performed over a 2‐year period. Patients with transsphincteric anal fistula who underwent SIFT‐IS were enrolled. The primary endpoint was the anal fistula healing rate at 16 weeks postoperatively. The secondary endpoints were healing time, postoperative complications and clinical continence status.

**Results:**

One hundred and fifty one patients were enrolled. Primary healing was accomplished in 129 patients (85%). There were 17 patients (11%) with a remnant fistula and five (3%) with a recurrence. The remnant fistulas healed spontaneously at more than 16 weeks postoperatively in seven patients. The median healing time was 6 (3–96) weeks. Surgical intervention was required in seven patients with a remnant fistula and four with recurrence. At the final follow‐up, the wounds had healed in 148 patients (98%). No significant postoperative complications or incontinence were observed.

**Conclusion:**

Subcutaneous incision of the fistula tract and internal sphincterotomy is a promising surgical option for transsphincteric anal fistulas, with a satisfactory healing rate.

**What does this paper add to the literature?**

The markedly deep crypts in the primary opening of an anal fistula may be one of the causes of the disease. Subcutaneous incision of the fistula tract and internal sphincterotomy is a procedure to eradicate the deep crypts and has a satisfactory healing rate for transsphincteric anal fistulas.

## INTRODUCTION

The aetiology of anal fistulas is thought to be cryptoglandular infection [1]. Faecal contents enter the crypts; infection then begins in the anal glands and the infection progresses to the sphincter and adjacent adipose tissue. Ninety per cent of anal fistulas are attributed to cryptoglandular infection [1]. Based on this cryptoglandular infection theory, the principles of anal fistula treatment are to block the inflow of faecal contents to the fistula and eradicate the septic focus, including the infected anal gland. Fistulotomy is still the most reliable surgical procedure, achieving a healing rate of more than 90% [2, 3]. However, as fistulotomy has a risk of impairing sphincter function [3, 4], sphincter‐sparing procedures (SSPs) have been established, including the endorectal advancement flap technique [5, 6, 7] and ligation of the intersphincteric fistula tract (LIFT) [8]. These SSPs are also based on the theory that anal fistulas are caused by cryptoglandular infection.

The aetiology of anal fistulas is still not completely clarified. Perianal abscesses and fistulas are considered two sequential phases of the same anorectal infectious process. Perianal abscesses arise from cryptoglandular infections and represent the acute phase, while anal fistulas represent the chronic phase; however, perianal abscesses do not always progress to anal fistulas; the frequency ranges from 16% to 41% [9, 10, 11, 12, 13]. The cryptoglandular infection theory is unable to explain why some perianal abscesses develop into anal fistulas and others do not. This suggests that there are other factors that promote the progression of perianal abscesses to anal fistulas.

**FIGURE 1:**
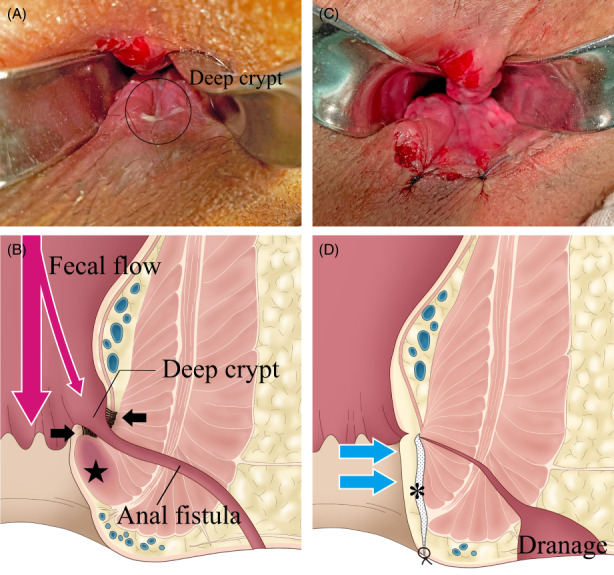
Preoperative (A, B) and postoperative (C, D) images. (A) A deep crypt is shown as a cave‐like dent. (B) Deep crypts are formed by the contraction of inflammatory fibrosis (➡). The internal sphincter around the crypt is hardened by inflammatory changes (★), which is considered to act as a kind of receptacle and make it easier for faecal contents to enter the crypt. (C) The deep crypt disappears. (D) Dissection of the fistula and fibrous tissue, and cutting of the fibrous internal sphincter lead to the flattening of the anal canal (blue arrows). The asterisk (*) indicates the dissecting layer.

The primary openings of anal fistulas comprise wide and thick pockets referred to as deep crypts (Figure 1A). The shape of these deep crypts may promote the entry of faecal contents from the primary opening and lead to persistent infection. Deep crypts are considered to cause anal fistulas in infants [14, 15], but there have been no reports of deep crypts as the aetiology of anal fistula in adults.

Clinical observations suggest that deep crypts are likely to be formed as the anorectal infection progresses from cryptoglandular infection to anal fistula. Contraction of the fibrous tissue attached behind the primary openings accentuates and opens the crypts to form deep crypts (Figure 1B). Pathological examination has also revealed inflammatory fibrosis in the subepithelial layer around anal fistulas [16]. Once deep crypts are created, they may promote continuous inflow of faecal contents into the fistula, causing chronic inflammation and preventing the fistula from healing. We considered that eradicating these deep crypts was the key to treating anal fistulas. Thus, we developed a surgical procedure consisting of two techniques, namely subcutaneous incision of the fistula tract (SIFT) and internal sphincterotomy (IS) (Video S1).

The aim of SIFT is to flatten deep crypts by dissecting fistula tracts and fibrous subepithelial tissue connecting the deep crypt, which prevents faecal contents from entering the internal openings. The role of IS is to restore the compliance and distensibility of the fibrotic internal sphincter, which complements the effects of SIFT.

The purpose of the current study was to assess the feasibility of SIFT‐IS for treating transsphincteric anal fistulas.

## METHOD

### Patients and methods

This retrospective study was conducted at the Coloproctology Center, Tokyo Yamate Medical Center, Japan, from January 2016 to December 2017. RS and KM worked there until March 2020. The patients provided written informed consent for the procedure, and the study was approved by the hospital's institutional review board.

Patients with transsphincteric fistula were included in this study. In accordance with the Parks classification [17], transsphincteric anal fistulas were defined as fistulas crossing both the internal and external sphincters. Transsphincteric anal fistulas were identified via digital examination and proctoscopy and confirmed by intraoperative findings. Magnetic resonance imaging was used to differentiate transsphincteric anal fistulas from complicated fistulas (suprasphincteric and extrasphincteric fistulas) when necessary. The following were excluded: patients with multiple fistulas, recurrent fistula, horseshoe fistula and fistulas associated with Crohn's disease.

The primary endpoint was the healing rate at 16 weeks postoperatively. Fistulas were considered healed when the surgical wound achieved complete epithelialization. Treatment failure was defined as a remnant fistula or recurrence. The presence of an unhealed wound at 16 weeks postoperatively was regarded as a remnant fistula. Recurrence was defined as the reappearance of local sepsis or fistula after primary wound healing had been observed. The secondary endpoints were healing time, postoperative complications and clinical continence status. Continence status was assessed based on interviews performed at each pre‐ and postoperative follow‐up medical examination.

### Operative technique

**FIGURE 2:**
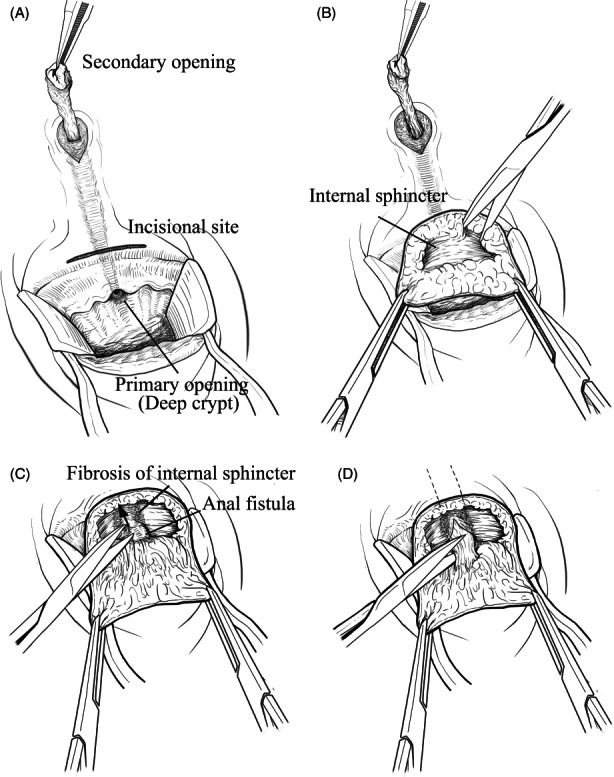
(A) The fistula is dissected from the secondary opening and then an incision is made on the lower edge of the anoderm. (B) The subepithelial layer is dissected through the incision. (C) Internal sphincterotomy is performed on the fibrotic internal muscle which is adjacent to the fistula. (D) The fistula is divided on the surface of the internal sphincter without ligation.

All patients were administered a laxative suppository (New Lecicarbon, Kyoto Pharmaceutical Industries) preoperatively. Surgery was performed with the patient in the prone jackknife position under spinal anaesthesia. The buttocks were stretched laterally using adhesive tape. After identification of the secondary opening, an encircling incision was made around the secondary opening and diathermy dissection was performed along the fistula to the lateral surface of the external sphincter where the fistula penetrated (Figure 2A). A 3 cm arc‐shaped incision was then made in the orthogonal direction above the fistula at the lower edge of the anoderm. Diathermy and blunt submucosal dissection were performed along the surface of the internal sphincter toward the cranial side (Figure 2B). Meticulous dissection was necessary to prevent injury to the anoderm. The fistula was identified as a white fibrotic tract running in the dissected layer. The fibrotic subepithelial tissue around the fistula lining the deep crypt was dissected along the surface of the internal sphincteric plane. We found that the lower edge of the internal sphincter through which the fistula penetrated appeared fibrous and hardened (Figure 1B). Shallow and sharp sphincterotomy was performed at the affected point using a scalpel or scissors (Figure 2C); in our experience, this seems to restore the compliance and distensibility of the internal sphincter. The fistula was encircled and divided at the level of the internal sphincter plane without ligation (Figure 2D). No treatment was performed on the primary opening in the bowel lumen. The distal fistula running from the outside of the external sphincter to the external opening was removed. A cone‐shaped drainage tract was created at the excised portion of the distal fistula. The fistula tract running from the internal sphincter to the external sphincter was not treated. The incision at the lower edge of the anoderm was loosely approximated. The deep crypt recognized preoperatively was flattened after surgery (Figure 1C,D).

All patients were administered oral cefcapene pivoxil (300 mg/day for 3 days) postoperatively and were discharged within 3 days postoperatively. Follow‐up examinations were performed every 2–4 weeks until complete wound healing was achieved. Patients with follow‐up of less than 16 weeks and without confirmed wound healing were treated as drop‐outs. The assessed variables included healing time, recurrence, clinical continence status and any other associated morbidities.

## RESULTS

**FIGURE 3:**
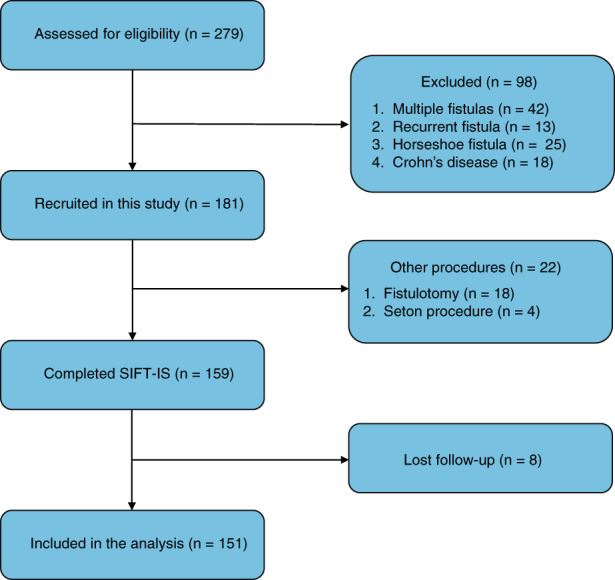
Flow diagram of patient screening (SIFT‐IS, subcutaneous incision of the fistula tract and internal sphincterotomy).

There were 1720 patients with anal fistula during the study period. Six hundred and ninety one patients were assigned to RS. Among these, 279 patients were diagnosed as having a transsphincteric fistula. SIFT‐IS was performed in 159 of 181 recruited patients. Other procedures were performed in 22 patients who had a very short fistula tracts or immature fistula. Eventually, 151 patients were included in this study. The flow diagram of screened patients is shown in Figure 3. The median patient age was 37 (13–88) years, and there were 129 men (85%) and 22 women (15%). Forty six (30%) patients had previously undergone incision and drainage for an anal abscess before formation of the anal fistula. Nine patients (6%) had a history of surgery for an anal fistula at a different site to the present fistula. Patient characteristics are shown in Table 1.

**TABLE 1:** Characteristics of enrolled patients who underwent subcutaneous incision of fistula tract and internal sphincterotomy (SIFT‐IS)

Patient characteristic	*n* (%)
Age (years), median (range)	37 (13–88)
Sex	
Male	129 (85)
Female	22(15)
History of previous incision and drainage	46 (30)
Location of the anal fistula	
Anterior	60 (40)
Lateral	80 (53)
Posterior	11 (7)
Previous perianal surgery	
Haemorrhoid surgery	5 (3)
Anal fistula surgery	9 (6)

*Note*: Data are presented as *n* (%) unless stated otherwise.

No patients received seton placement prior to SIFT‐IS because we only perform seton placement for patients with a complicated fistula or Crohn's disease.

### Outcomes of SIFT‐IS

Primary healing was achieved in 129 patients (85%). The median duration of follow‐up was 10 (3–131) weeks. There were 17 patients (11%) with a remnant fistula and five (3%) with a recurrence.

Among the patients with a remnant fistula, the wound healed spontaneously without any surgical intervention within 24 weeks in six patients and at 96 weeks in one patient. Thus, 136 patients (90%) were healed by SIFT‐IS without other procedures. The median healing time for SIFT‐IS including the patients who were healed after 16 weeks postoperatively without further surgical intervention was 6 (3–96) weeks. Seven patients required surgical intervention for a remnant fistula. Three patients are still being followed up.

**FIGURE 4:**
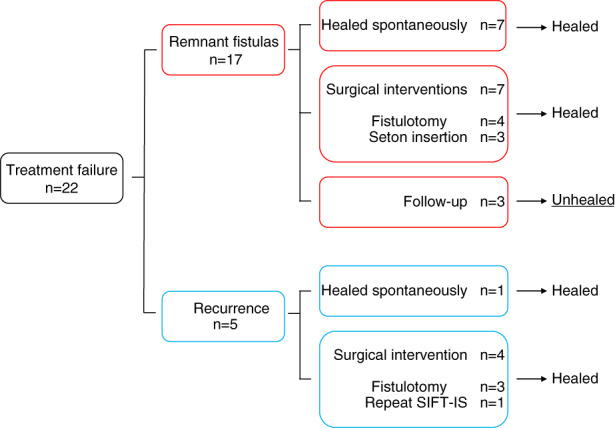
Flow diagram showing the outcomes of patients who did not heal after subcutaneous incision of the fistula tract and internal sphincterotomy (SIFT‐IS).

Five patients developed recurrence at 20–47 weeks after the primary surgery. Four patients required surgical intervention, while one healed spontaneously. A flow chart of the patients with treatment failure is shown in Figure 4.

Three of the total of 11 patients who needed further surgical intervention had a transsphincteric fistula that converted into a subepithelial fistula; these were treated via fistulotomy under local anaesthesia without readmission. The remaining eight patients had a residual transsphincteric fistula tract; of these, three patients underwent fistulotomy, one underwent repeat SIFT‐IS and four had a tight seton inserted. All patients with treatment failure who received surgical intervention were successfully cured. The median healing time after the surgical interventions was 11 (5–111) weeks. Complete healing of the fistula was finally accomplished in 148 patients (98%). The outcomes are summarized in Table 2.

**TABLE 2:** Operative findings and outcomes (*N* = 151)

Finding/outcome	*n* (%)
Follow‐up duration (weeks), median (range)	10 (3–131)
Healing time (weeks), median (range)	6 (3–15)
Primary healing at 16 weeks	129 (85)
Treatment failure	
Remaining unhealed wound	17 (13)
Recurrence	5(3)
Wound finally healed	148 (98)

*Note*: Data are presented as *n* (%) unless stated otherwise.

### Postoperative complications

Four patients had postoperative bleeding from the drainage wound that required haemostasis with bipolar forceps; none of these patients experienced changes in vital signs or required infusion or blood transfusion. There were no postoperative complications related to the surgical wound, such as abscess formation, severe pain or dermatitis. No patient experienced changes in their clinical continence.

## DISCUSSION

SIFT‐IS is a novel surgical procedure for anal fistula. The essential feature of SIFT‐IS is to eliminate the structural abnormalities of primary openings that cause persistent infection of the anal fistula. Our study showed that SIFT‐IS achieved primary healing in 85% of patients.

The Parks classification categorizes anal fistulas as intersphincteric, transsphincteric, suprasphincteric or extrasphincteric [1]. We focused on transsphincteric fistulas because these are the most common fistula type. Fistulotomy is the most reliable procedure for treating transsphincteric fistula in terms of healing rate, but postoperative incontinence occurs in up to 54% of cases [3, 4]. The risk of postoperative incontinence depends on the amount of sphincter muscle involved in the fistula [4, 18, 19]; nevertheless, incontinence, including mild continence disorders, was observed in 24% even in patients with a low fistula (less than one‐third of the external sphincter division) [19]. It is necessary to consider a less invasive procedure for sphincter muscles in the treatment of transsphincteric anal fistula.

The primary healing rate with SIFT‐IS was 85% at 16 weeks, reaching 89% at 24 weeks without further surgical intervention. These results are comparable to the SSPs adopted by many surgeons. Previous studies have reported healing rates of 66%–83% after the endoanal advancement flap procedure [20, 21, 22] and 68%–88% after LIFT [23, 24, 25, 26]. Our results suggest validity of the concept that SIFT‐IS restores the structure of the primary opening, leading to healing of the anal fistula. We started to develop the SIFT‐IS procedure in 2007. The tract stumps were initially supposed to be ligated, but we found that anal fistulas healed without ligation in patients in whom the fistula tracts running through the subepithelial layer were too thin and fragile to be ligated. Thus, we stopped ligating the fistula tracts and verified that they still healed. In anal fistula treatment, it has previously been considered necessary to close the primary opening where the faecal contents enter. However, we assume that flattening the primary opening prevents faecal contents from flowing into the crypt, which performs the same role as primary opening closure. Consequently, ligation of the fistula tract is unnecessary.

Our study may provide new insights into the aetiology of anal fistula. Cryptoglandular infection is considered the first step in the development of a perianal abscess, which is the preliminary stage of anal fistula formation. However, the cryptoglandular infection theory is not sufficient to explain the mechanism by which a perianal abscess transforms into an anal fistula because the majority of perianal abscesses will not develop into anal fistulas [9, 10, 11, 12, 13]. The largest and the most recent series demonstrated that anal fistulas occurred in only 16% of patients with perianal abscesses [13]. Therefore, there must be other factors that allow persistent infections and promote the formation of an anal fistula from a perianal abscess. We speculate that the transformation of a perianal abscess to an anal fistula is contingent on whether the inflammation of the perianal abscess spreads to the crypt and whether the crypt becomes deep. Once deep crypts are formed, they may enable persistent infection and promote the formation of an anal fistula. The development of an anal fistula may require both cryptoglandular infection and deep crypt formation.

Comparing existing procedures, we believe that SIFT‐IS has beneficial clinical characteristics owing to the dissection in the subepithelial layer. Fistulotomy is the most prevalent procedure for transsphincteric fistulas, but there are risks associated with impaired anal continence owing to the division of the internal sphincter [3, 4, 18, 19] SIFT‐IS may overcome the postoperative incontinence associated with fistulotomy because the SIFT‐IS approach is less invasive with regard to the internal sphincter.

LIFT is a recent and widely performed SSP in which the anal fistula is divided and ligated in the dissected intersphincteric layer [8]. LIFT is associated with several risks while dissecting the intersphincteric layer that can lead to impaired anal function because the conjoined longitudinal muscle runs between the internal and external sphincters.

Contraction of the longitudinal muscle plays a role in shortening and widening of the anal canal during the process of defaecation [27], and LIFT may harm the function of the muscle. The impairment of fine nerve networks and generation of scar tissue in the intersphincteric layer in LIFT may also interfere with the coordinated movement of sphincteric muscles. Postoperative evaluation of incontinence using surveillance of Cleveland Clinic Florida Faecal Incontinence scores detected minor incontinence after LIFT [28, 29]. In contrast, since SIFT‐IS does not affect the intersphincteric layer, it would not be expected to damage the coordinated movement of the anal sphincter complex.

In the present study, no patient had faecal incontinence. SIFT‐IS involves cutting the internal sphincter, but IS seems to have only a minor effect on internal sphincter function because the cutting length of the IS is less than 1 cm and the depth is very shallow. Furthermore, IS restores the compliance and distensibility of the fibrotic internal sphincter, which may even be beneficial to anal function. However, the incidence of faecal incontinence based on the medical records may have been underestimated. Validated continence scoring systems and anal manometry are required to accurately evaluate anal competence after SIFT‐IS.

Another beneficial characteristic of SIFT‐IS is that treatment failure comprised downstaging from a transsphincteric to a superficial fistula. Three of 11 (27%) patients with treatment failure who needed surgical intervention had a subepithelial fistula after SIFT‐IS. In contrast, patients with treatment failure after LIFT reportedly have downstaging of a transsphincteric fistula into an intersphincteric fistula [30, 31], probably owing to breakdown of the fistula stump after the primary surgery and the formation of a drainage route from the primary opening to the dissected surgical layer. The downstagings after both SIFT‐IS and LIFT are favourable failure patterns because the downstaged fistulas can be treated with simple procedures, such as fistulectomy, that are less invasive with regard to anal function. Furthermore, the degree of downstaging in SIFT‐IS treatment failure is greater with results that are easier to treat than after LIFT. As a superficial fistula does not include the sphincter muscles, it can be cured simply by cutting the epithelium under local anaesthesia without readmission. Conversion from transsphincteric to superficial fistula in SIFT‐IS is a characteristic and acceptable failure pattern.

In the present study, prophylactic antibiotics were administrated postoperatively based on traditional practice. We currently only administer prophylactic antibiotics preoperatively in accordance with the Japanese guidelines for antimicrobial prophylaxis published in 2016 [32].

This is the first study of SIFT‐IS, which was conducted in patients with a simple transsphincteric fistula. We are now investigating the efficacy of SIFT‐IS in patients with complicated fistulas, namely multiple, horseshoe and recurrent fistulas. Currently, we are aware that the indications for recurrent fistulas with severe fibrosis around the primary opening should be carefully considered because subcutaneous dissection could involve technical difficulties leading to mucosal injury and failure to eradicate the deep crypts. We consider that deroofing procedures from the primary opening might be more appropriate than SIFT‐IS. Recent deroofing procedures from the primary opening to the intersphincteric layer without division of the external sphincter, such as TROPIS (transanal opening of the intersphincteric space) [33] and Bastawrous‐modified LIFT [34], might be reasonable in terms of safety, curability and preservation of anal function. SIFT‐IS, which does not include a deroofing procedure, might be beneficial for postoperative continence status and a short healing time of the surgical wound. However, careful case selection would be necessary to adapt SIFT‐IS for complicated fistulas with severe fibrosis.

The present study has several limitations. First, structural abnormalities of the primary opening, which may be attributed to fibrosis caused by cryptoglandular infection, were assessed through our clinical and surgical observations. Histopathological examination is necessary to confirm these findings. Second, patient selection was non‐consecutive and the outcomes were evaluated by the attending surgeons. Thus, this study might include selection and information bias. Third, in this study the follow‐up examinations were complete when operative wound healing was confirmed. This contributed to the short median follow‐up period (10 weeks) and might have caused underestimation of the recurrence rate. For the future we plan to assess an accurate recurrence rate with an adequate follow‐up period and identify the risk factors for remnant fistula and recurrence.

## CONCLUSION

SIFT‐IS is a novel and promising procedure for treating anal fistula. We hope that our research will contribute to providing new insights into the incompletely understood aetiology of anal fistula.

## AUTHOR CONTRIBUTIONS

RS developed the surgical procedure, collected the data and contributed to the design of this study. MK analysed the data and wrote the manuscript. KM collected and interpreted the data and prepared the figures and tables. IK contributed to the interpretation of the results and critical revision of the manuscript. All authors discussed the results and approved the final version of the manuscript.

## Funding information

This research received no specific grant from any funding agency in the public, commercial or not‐for‐profit sectors.

## CONFLICT OF INTEREST

The authors have no conflicts of interest relevant to the content of this manuscript.

## ETHICS APPROVAL

The patients provided written informed consent for the procedure, and the study was approved by the Institutional Review Board of Tokyo Yamate Medical Center.

## PATIENT CONSENT STATEMENT

Informed consent was obtained from all individual participants included in the study.

## PERMISSION TO REPRODUCER MATERIAL FROM OTHER SOURCES

None.

## Supporting information


Video S1

## Data Availability

The data that support the findings of this study are available on request from the corresponding author. The data are not publicly available due to privacy or ethical restrictions.
